# Blood Type as a Potential Predictor of Hemorrhagic Risk in Patients Undergoing Partial Hepatectomy for Colorectal Liver Metastasis

**DOI:** 10.3390/jcm14113905

**Published:** 2025-06-02

**Authors:** Wisam Assaf, Esther Kazlow, Max Rowe, Reem Gawi, Aasem Abu Shtaya, Hanin Barsha, Yakir Segev, Riad Haddad, Ahmad Mahamid

**Affiliations:** 1 Department of Obstetrics and Gynecology, Lady Davis Carmel Medical Center, Haifa 3436212, Israel; haninba@gmail.com (H.B.); yakirse@gmail.com (Y.S.); 2 The Ruth and Bruce Rappaport Faculty of Medicine, Technion, Israel Institute of Technology, Haifa 3525433, Israel; ekazlow@gmail.com (E.K.); reem.gawi@mail.huji.ac.il (R.G.); dr.riad.haddad@gmail.com (R.H.); mahamidam@yahoo.com (A.M.); 3 Department of Surgery, Lady Davis Carmel Medical Center, Haifa 3436212, Israel; 4 Department of Gastroenterology, Lady Davis Carmel Medical Center, Haifa 3436212, Israel

**Keywords:** blood type, bleeding, partial hepatectomy, colorectal cancer

## Abstract

**Background:** Hepatic resection is performed for liver lesions and requires careful preoperative planning to minimize bleeding. Blood type O, associated with lower von Willebrand factor (vWF) levels, may increase bleeding risk. This study investigates the relationship between the ABO blood type and perioperative bleeding in partial hepatectomy for colorectal liver metastases (CRLMs). **Methods:** Out of 563 patients who underwent hepatectomy, 135 cases were analyzed for CRLM at Carmel Medical Center (2013–2023). Patients were categorized into blood type O (61 patients) and non-O (74 patients) groups. Data on perioperative hemoglobin levels, blood loss, coagulation parameters, transfusion needs, and complications were assessed using χ^2^, *t*-tests, and ANOVA (*p* < 0.05). **Results:** No significant differences were observed for estimated blood loss (474.3 ± 696 mL for O vs. 527.8 ± 599 mL for non-O; *p* = 0.29), intraoperative hemoglobin drop (*p* = 0.613), or transfusion rates (24.59% for O vs. 28.37% for non-O; *p* = 0.698). Although non-O patients had a higher postoperative INR (*p* = 0.035), this did not correlate with increased bleeding or transfusion needs. **Conclusions:** Blood type O does not significantly affect perioperative bleeding or transfusion requirements in partial hepatectomy for CRLM. Further research is needed to better understand the significance of the ABO blood type.

## 1. Introduction

Hepatic resection is often indicated for treating a range of both benign and malignant liver conditions. Planning for this procedure requires the assessment of numerous factors including the location of the lesion within the liver, the patient’s native anatomy, and ensuring sufficient remaining liver tissue post resection (referred to as adequate future liver remnant (FLR)) [1]. Perioperative outcomes for hepatic resection have improved over the years due to better surgical techniques, better strategies for controlling perioperative bleeding, and improved postoperative care in intensive care units [1,2,3].

The liver is anatomically divided into two main lobes, and further segmented into eight regions based on vascular supply and bile duct distribution [4]. Additionally, the liver plays numerous crucial metabolic roles, and it produces bile to aid in digestion; processes nutrients absorbed from the digestive tract; stores glycogen, vitamins, and minerals; regulates blood sugar levels; and synthesizes plasma proteins such as albumin and clotting factors [4,5].

The primary reason for hepatic resection is typically the presence of a primary or secondary malignant tumor in the liver. However, there are instances where benign liver conditions may also necessitate hepatic resection [6,7].

The incidence of post-hepatectomy coagulopathy is difficult to determine [8]. Numerous factors contribute to coagulopathy after hepatic resection including hemodynamic instability, intraoperative blood loss, preexisting hepatic dysfunction, acute liver injury, the failure of the remaining liver tissue (FLR), and hypothermia [9,10].

Some researchers have suggested a potential link between major blood types and the propensity for perioperative bleeding [11]. In particular, the O blood type has been implicated as a potential risk factor for bleeding in various surgical procedures such as vaginal hysterectomy and tonsillectomy [12,13,14].

von Willebrand factor (vWF) is a glycoprotein that plays a crucial role in primary hemostasis by facilitating the adhesion of platelets to collagen and by stabilizing factor VIII within the intrinsic pathway of the coagulation cascade. Structural similarities between specific polysaccharide side chains on the vWF molecule and A and B blood group antigens may slow down vWF metabolism and degradation [15]. As a result, individuals with blood types A, B, or AB may exhibit higher plasma levels of vWF, potentially enhancing their coagulation capacity [16]. In contrast, those with blood type O—who do not express A or B antigens—often have reduced vWF levels, which could increase their risk of bleeding [14,17,18,19].

The relationship between blood type, vWF, and operative bleeding is influenced by vWF levels, which are lower in individuals with blood type O (25–35% less than non-O types) [20,21]. This reduction may increase the risk of bleeding during and after surgery. Studies have shown that blood type O patients undergoing total hip arthroplasty tend to have higher postoperative bleeding volumes [22]. In pediatric tonsillectomy patients, lower vWF levels were observed in blood type O, though without a significant increase in postoperative hemorrhage [23]. In trauma cases, blood type O has been linked to hyperfibrinolysis and a higher need for massive transfusion [20]. However, surgeries such as coronary artery bypass grafting and acute type A aortic dissection have not shown significant differences in bleeding risks between blood type O and non-O patients [24].

However, the impact of blood type on hemorrhagic risk following perioperative partial hepatectomy surgery remains largely unexplored. Our study examines the impact of blood type on the potential for bleeding during and following partial hepatectomy.

## 2. Material and Methods

Patients who underwent partial hepatectomy for CRLM between January 2013 and September 2023 were identified from the surgical databases at Carmel Medical Center (Haifa, Israel), a university-affiliated tertiary care hospital with a substantial hepato-biliary unit. The data were collected from the Chameleon database, a comprehensive electronic medical record system, which includes detailed patient information and perioperative data. The study was conducted in accordance with the Declaration of Helsinki and Good Clinical Practice Guidelines, and was approved by the institutional review board (IRB) of Carmel Medical Center. Our review included the examination of patient demographics, detailed surgical history, pathology results, and follow-up from the medical/oncology team. All patients were evaluated for surgery by an attending anesthesiologist and approved for surgery by a multidisciplinary team. All patients were informed about the procedure, including risks and benefits. Written consent for surgery was obtained from all patients. Due to the retrospective nature of the study, informed consent from patients was waived by the Institutional Review Board Committee. After discharge, all the patients were followed by our multidisciplinary team during the first month post surgery, every 4 months for the first 2 years, and subsequently twice a year.

### 2.1. Study Population 

Patients aged 18 to 90 years who underwent partial hepatectomy for CRLM with an adenocarcinoma histologic type as a single surgery (no patients underwent colectomy during the same operation) and had documented ABO blood types in the hospital’s database were included in the study. All partial hepatectomies for CRLM included in this study were performed via an open surgical approach by the same experienced hepatobiliary surgical team. Exclusion criteria included the use of anticoagulation or antiplatelet medications that were not discontinued before surgery.

### 2.2. Variables

Data collected for each patient included demographic information, past medical and surgical history, ABO blood type, perioperative hemoglobin levels, platelet count, international normalized ratio (INR), vitamin D levels, albumin levels, cancer antigen 19-9 (CA 19-9) levels, and carcinoembryonic antigen (CEA) levels.

FLR volume was assessed using 3D reconstruction of multiphasic Computed Tomography (CT) scans and expressed as a percentage of total liver volume (TLV), excluding tumors. This National Comprehensive Cancer Network (NCCN)-recommended method sets FLR thresholds at ≥20% for normal livers and ≥30% for chemotherapy-injured livers. To minimize post-hepatectomy liver failure, we included only patients with FLR ≥ 30% [25,26,27].

Estimated blood loss (EBL) during surgery was defined as the volume of blood suctioned from the abdominal cavity during the procedure. Operative time was defined as the time elapsed from the initial incision until closure. In our study, blood pressure was maintained within the lower permitted variability range to minimize its impact on blood loss. Postoperative hospital stay was defined as the number of hospitalized days from the day of operation until the day of discharge, inclusive. Tumor size and resection margins were determined based on pathology reports taken from the permanent sections of tissue samples. Complications were defined as any unexpected event that deviated from a normal recovery course, occurring within 30 days of surgery. The severity of complications was graded using the Clavien–Dindo scoring system [28].

### 2.3. Group Distribution

Patients were categorized into two groups based on blood type—O and non-O (A, B, or AB). The primary outcome assessed was perioperative bleeding which was evaluated by comparing preoperative hemoglobin levels, postoperative hemoglobin levels, and the lowest hemoglobin level recorded during hospitalization (ΔHemoglobin). To control for potential confounding factors, platelet counts and coagulation parameters were analyzed using the same approach.

### 2.4. Sample Size Calculation and Power Analysis

Based on the prevalence of blood types in the general population (O = 35%, A = 38%, B = 18%, AB = 7.8%) and a confidence level of 95% with a power of 80%, a minimum of 128 patients were required to detect a 0.6 standard deviation difference in hemoglobin levels between blood type O and non-O patients.

### 2.5. Statistical Analysis

All statistical analyses were performed using IBM statistics (SPSS) vs. 24. Comparisons between categorical variables were made using the χ^2^ test or Fisher’s exact test, while continuous variables were compared using the *t*-test or ANOVA. A stepwise logistic regression model was used to estimate the effect of each variable. A *p*-value of <0.05 was considered statistically significant.

Prior to performing *t*-tests or ANOVA for continuous variables, such as hemoglobin levels, their distribution was assessed for normality and symmetry. This assessment included the visual inspection of Q-Q plots and the comparison of mean and median values. Following this assessment, data in the text are presented as mean ± standard deviation. For graphical representation in figures, median and interquartile range (IQR) are used to provide a robust visual summary.

## 3. Results

**Chart 1:**
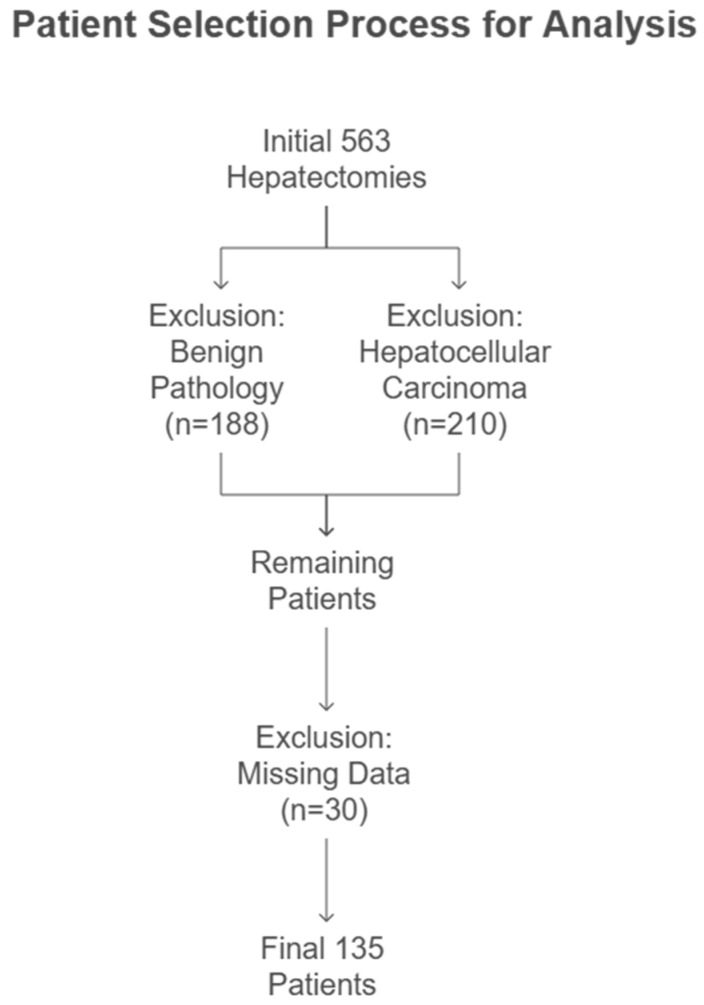
Patient selection process.

During the study period, 563 patients underwent partial hepatectomy for various conditions. Of these partial hepatectomies, 188 procedures were performed for benign conditions, while 210 were performed for hepatocellular carcinoma. To ensure a more homogeneous study population and minimize confounding factors related to coagulopathies and significant liver function abnormalities, we selected patients who underwent partial hepatectomy specifically for CRLM. The study cohort included 165 patients, of whom 30 (18%) were excluded due to missing information, mainly hemoglobin (HB) levels or blood type (Chart 1). Of the remaining 135 patients, 61 (45.19%) had the O blood type, while 74 (54.81%) had the A, B, or AB blood types. All patients underwent partial hepatectomy due to colorectal cancer; therefore, the Child–Pugh score was A for all patients. Characteristics of the study population (demographics and clinical covariates) are presented in Table 1.

**Figure 1:**
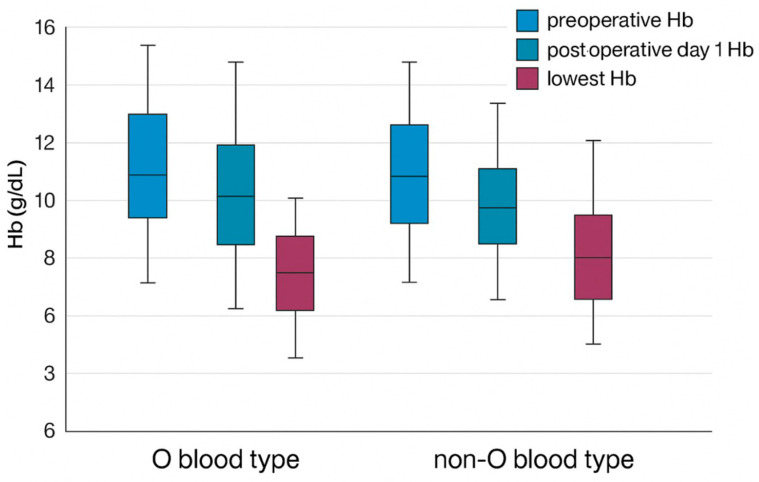
A comparison of hemoglobin levels between blood type O and non-O over a different time period. The black vertical lines represent the range, the colored boxes represent the 25th and 75th percentiles of the IQR, and the black lines inside represent the median. There was no statistical difference between O blood type and non-O blood type in preoperative, first postoperative, and lowest hemoglobin values, with p-values of 0.538, 0.517, and 0.128, respectively.

The mean preoperative Hb level was 12.7 ± 1.5 g/dL for individuals with the O blood type and 12.0 ± 1.4 g/dL for individuals with a non-O blood type (*p* = 0.538) (Figure 1). There was no significant difference in preoperative platelet count, with a mean of 222.5 ± 59.6 × 10⁹/L for individuals with the O blood type and 211.9 ± 75.1 × 10⁹/L for individuals with a non-O blood type (*p* = 0.193). Similarly, the international normalized ratio (INR) did not differ significantly between the two, with values of 1.08 ± 0.41 for individuals with the O blood type and 1.06 ± 0.17 for individuals with a non-O blood type (*p* = 0.406) (Table 2).

**Figure 2:**
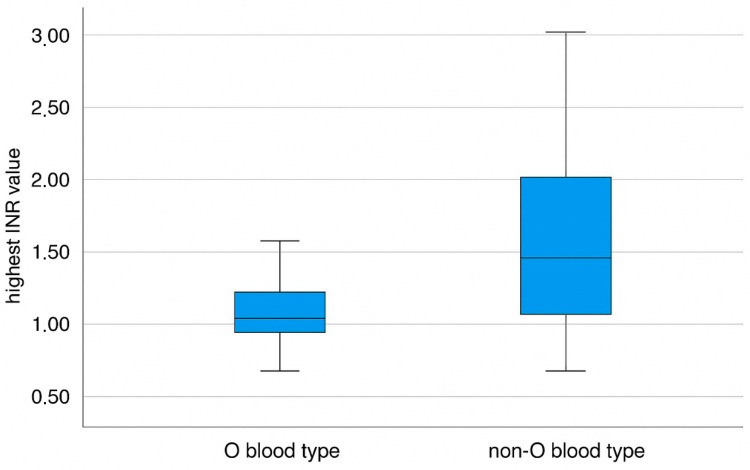
A comparison of highest INR value between blood type O and non-O. The black vertical lines represent the range, the boxes indicate the 25th and 75th percentiles of the IQR, and the black lines inside the boxes represent the median. There was a statistical difference between the O blood type and non-O blood type in the highest INR value, with a *p*-value of 0.035.

**Figure 3:**
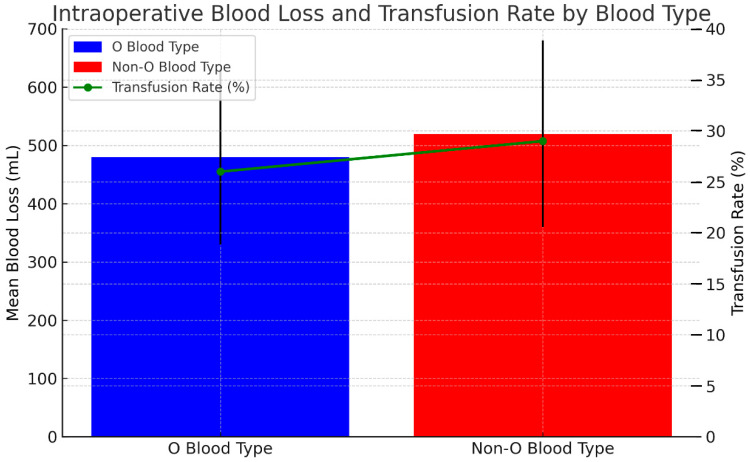
Intraoperative Blood Loss and Transfusion Rate by Blood Type.

The mean estimated intraoperative blood loss was 474.3 ± 696 mL for the O blood type group and 527.8 ± 599 mL for the non-O blood type group (*p* = 0.29). In total, 24.59% of the patients in the O blood type group needed blood transfusions during surgery, and 28.37% of the non-O type blood group required intraoperative blood transfusions as well, *p* = 0.698 (Table 1, Figure 2 and Figure 3).

There was no significant difference in the intraoperative Hb drop (calculated by subtracting Hb on postoperative day 1 (POD-1) from pre-operative Hb) between the two blood types. The intraoperative Hb drop was 1.67 ± 1.46 g/dL for individuals with the O blood type and 1.74 ± 1.47 g/dL for individuals with a non-O blood type (*p* = 0.613). Furthermore, there was no significant difference in the postoperative Hb drop (calculated by subtracting the lowest Hb recorded during admission on any POD from Hb on POD-1) between the two blood types. The postoperative Hb drop was 1.56 ± 0.97 g/dL for individuals with the O blood type and 1.30 ± 1.2 g/dL for individuals with a non-O blood type (*p* = 0.349), (Table 3, Figure 3).

Even after excluding patients who received intraoperative blood transfusions, there was no difference in intraoperative (*p* = 0.522) or postoperative (*p* = 0.354) HB drop between the groups (Table 3). Five patients in the O blood type group experienced a HB drop of more than 3 g/dL, compared to six in the non-O blood type group (*p* = 0.985).

Patients with a non-O blood type exhibited a statistically significant higher postoperative INR compared to those with the O blood type (1.66 ± 0.68 vs. 1.41 ± 0.44, respectively; *p* = 0.035) (Figure 2). However, this difference did not translate into increased bleeding, greater transfusion requirements, or other complications (Table 2).

A comprehensive analysis revealed no statistically significant differences between the O and non-O blood type groups across multiple clinical and demographic parameters. Specifically, the two groups exhibited comparable age distributions, medical histories, and pre-operative biochemical markers, including vitamin D and albumin levels, as well as tumor markers such as CA 19-9 and CEA. Additionally, there were no significant variations in tumor burden, as indicated by the size of the largest tumor, or in disease recurrence rates. Surgical factors, including operation duration and the extent of liver resection, were also similar between the groups. Furthermore, postoperative outcomes, such as the overall complication rate, the severity of complications, and the necessity for blood transfusion after surgery, did not differ significantly. These findings suggest that blood type does not appear to be a determining factor influencing these perioperative and oncological outcomes (Table 1).

## 4. Discussion

The association between blood type and perioperative bleeding during and after partial hepatectomy is an area of research that is largely unexplored. In this study, we evaluated a cohort of patients undergoing partial hepatectomy for CRLM and found that blood type O was not associated with increased intraoperative or postoperative bleeding. The main result of the study is that there is no significant difference in hemoglobin drop between patients with O and non-O blood types during the perioperative period. This suggests that blood type does not influence perioperative hemoglobin changes. The blood type distribution among our patients aligns with that of white, non-Hispanic Americans, with 45% of the patients having blood type O [29] but differs from the Israeli population, where the prevalence of blood type O is 35% [30]. This discrepancy may be due to reported links between certain diseases and malignancies with specific blood types—gastric cancers are more common in individuals with blood type A, gastric and duodenal ulcers are more frequent in those with blood type O, and pancreatic cancers are more prevalent in non-O blood types (A, AB, or B) [31,32]. However, no known correlation exists between blood type and colorectal cancer or liver metastasis. Further research on this topic is warranted.

The mean intraoperative estimated blood loss as well as pre- to postoperative hemoglobin did not significantly differ between the O and non-O blood type groups. Also, there was no difference between the groups in the number of packed red blood cell transfusions or in the percentage of patients who needed blood transfusions.

Individuals with blood type O have a distinct coagulation profile compared to those with other blood types, particularly due to lower levels and lower activity of vWF [33,34]. Since any quantitative or qualitative disruption of vWF can increase bleeding risk [35], understanding the factors influencing its levels is important. The half-life and plasma concentration of vWF varies widely among individuals, with one explanation being the interaction between the ABO blood types and vWF levels [14,34,35,36,37].

The vWF molecule contains side-chain oligosaccharides that resemble A and B blood type antigens, which are thought to reduce its degradation rate in the bloodstream [16,21]. This may explain why individuals with blood types A, B, and AB have, on average, 25% higher vWF levels than those with blood type O [33,37,38]. Supporting a genetic link, a large twin study by Orstavik et al. [39] found that 66% of the variability in plasma vWF levels is genetically determined, with ABO blood type accounting for 30% of this genetic component. Similarly, a large study of 1117 healthy individuals by Gill et al. [34] demonstrated that plasma vWF levels were lowest in blood group O and highest in group AB subjects.

Beyond bleeding risk, numerous studies indicate that the ABO blood group system influences hemostasis, and is an important predictor of thromboembolic events. For example, a large study (*n* = 71,729) found blood types A and AB conferred a higher risk of venous thromboembolism during pregnancy/puerperium compared to type O [40], while two other cohorts identified a relative risk of 1.7 to 2.1 for venous thromboembolism in non-O carriers [41,42]

Regarding hemorrhagic risk specifically, relatively few studies have examined the association with blood type O, yielding largely contradictory findings. One study found type O patients to be more likely to experience secondary post-tonsillectomy bleeding [14]. Another observed a higher prevalence of blood type O among patients admitted for epistaxis compared to controls, suggesting this may be a risk factor risk for this condition [43]. Further supporting a potential link, a meta-analysis revealed a significantly higher prevalence of blood type O in patients with certain coagulopathies compared to controls [16] and another study linked type O with severe mucosal hemorrhage in orally anticoagulated patients [11]. In obstetric settings, large cohort studies reported slightly increased risks of postpartum hemorrhage [44] or peripartum blood loss [45] in women with blood type O.

Conversely, several studies have found no significant association between blood type and hemorrhagic risk, suggesting that factors other than the ABO blood group may play a more prominent role in determining bleeding tendencies [16,24]. Proponents of these findings argue that while blood type O is hypothesized to influence coagulation, the lack of a consistent link across diverse research settings suggests that genetic, environmental, or clinical factors may be more important in predicting hemorrhagic risk than blood type alone. Furthermore, it is possible that variability in study designs, patient populations, and methodologies could also contribute to these conflicting findings. One study of 877 patients who underwent primary, nonemergent coronary artery bypass surgery showed that patients with blood type O did not experience increased bleeding after surgery compared to controls [24].

Similarly, large studies investigating early postpartum hemorrhage [16] or intraventricular hemorrhage in low-birth-weight infants found no significant associations with blood type [46,47]. Additionally, recent findings showed no association between major or minor blood groups and the risk of early postpartum hemorrhage or the need for blood transfusion [48]. In line with these studies, our study examined a homogeneous patient population in which multiple demographic and clinical confounders were assessed and found not to differ significantly between groups. We similarly found no evidence that blood type influenced bleeding tendency, which stands in contrast to the hypothesis suggesting a hemostatic disadvantage among individuals with blood type O. This conclusion is further supported by our sensitivity analyses of hemoglobin drop in nontransfused patients (Table 3), which accounted for the potential confounding effect of transfusion practices and continued to show no significant differences between the blood groups.

The EBL in partial hepatectomy can vary significantly based on several factors including, the highly vascular nature of the liver, pre-existing liver dysfunction, and surgical technique [49]. For instance, a study by Helling et al. reported that in major liver resections, the EBL could be as high as 7692 ± 3848 mL in cases with significant bleeding complications, while it was 1359 ± 514 mL in cases with less severe bleeding [50].

Another study by McNally et al. found that median blood loss for hepatic resections, including partial hepatectomies, was reported to be 782 mL [51] for laparoscopic liver resections, which are often associated with less blood loss compared to open procedures; the median blood loss was reported to be 120 mL, although this can vary significantly depending on the complexity of the surgery and the specific segments resected [52].

Although we examined several key factors that can influence bleeding—such as the size of the largest lesion, extent of liver resection, platelet count, and INR—and all surgeries were performed by the same hepatobiliary team to minimize variability in surgical techniques, other patient-specific factors like platelet function and vWF levels and activity were not assessed. These variables are not routinely tested before surgery and were beyond the scope of our retrospective study. As a result, the inability to account for these unmeasured factors may limit the reliability of blood type as a predictor of hemorrhagic risk. The complex interplay of these variables means that while blood type might play a role, its predictive value could be overshadowed by other, more influential determinants of bleeding. Additionally, the heterogeneity in the clinical presentation and management of patients undergoing partial hepatectomy further complicates the use of blood type as a standalone predictor of bleeding risk, making it less useful in guiding perioperative decision-making.

### Strengths and Limitations

The study’s main strengths include the comprehensive demographic and medical data which were entered electronically in real-time and can therefore be assumed to be accurately documented. Another strength of the study is that we examined several confounding factors that can potentially influence intraoperative or postoperative bleeding, including preoperative platelet count, INR, number of liver metastases, size of the largest tumor, operation duration, and extent of liver resection. Also, we examined factors that could affect hemoglobin levels, such as the number of blood transfusions, the number of transfused blood units, and surgical complications (assessed by the Clavien–Dindo scoring system). To accurately measure surgical bleeding, we calculated the decrease in hemoglobin levels between the first postoperative day and the lowest hemoglobin level during admission, alongside comparisons of platelet counts and INR on the same days.

This study is subject to several limitations that warrant consideration. The retrospective nature of our analysis inherently restricts the certainty of its conclusions and means that transfusion triggers were not uniformly mandated but based on clinical judgment; this potential variability was partially addressed by our sensitivity analyses in non-transfused patients. The single-center design may also limit the generalizability of our findings. Furthermore, the potential for some patients to have received preoperative chemotherapy, which can induce coagulopathy, may have introduced confounding variables affecting bleeding risk. The relatively high proportion (one-quarter) of patients requiring blood transfusions could also have limited the statistical power to discern significant differences within these specific subgroups.

A central limitation is the unavailability of data on key hemostatic modifiers. Specifically, von Willebrand factor (vWF) and Factor VIII levels, as well as platelet function, are not part of routine preoperative testing at our institution and were therefore not analyzed. This precluded a direct assessment of the biological link between ABO blood group, individual hemostatic function, and bleeding severity, thereby limiting the strength of any causal inferences.

Consequently, while blood type O is associated with lower vWF levels, our finding that it did not significantly increase perioperative bleeding or transfusion requirements in this cohort may be influenced by several factors. The multifactorial nature of perioperative hemostasis means that any subtle influence of vWF variations related to the ABO type could be mitigated or overshadowed. In particular, the meticulous surgical techniques employed by our specialized hepatobiliary team, the standardized use of low central venous pressure (CVP) anesthesia aimed at minimizing intraoperative blood loss, and potentially institutional transfusion thresholds, all contribute to minimizing overall bleeding. These highly effective contemporary practices might mask more subtle differences attributable solely to the blood group. Thus, the predictive value of blood type alone in this complex surgical context appears limited when considering these unmeasured biological variables and standardized clinical management strategies aimed at optimizing patient outcomes.

Given the limited and varying data currently available in the literature, we recommend further investigation into the role of blood groups as a predictor of perioperative hemorrhagic risk in patients undergoing partial hepatectomy, particularly in those with colorectal liver metastases, using larger patient cohorts. Measuring serum vWF concentrations in these patients could provide valuable insights and should ideally be included in such studies. While current evidence does not support using blood type to guide pre-operative cross-match strategies, prospective assessment of vWF levels may offer more meaningful predictive value. If blood groups or vWF levels are found to play a significant role, targeted strategies could be developed to identify patients at higher risk for perioperative bleeding, thereby improving preoperative planning, patient counseling, and surgical management to minimize bleeding complications.

## Figures and Tables

**Table 1:** Demographics and Baseline Characteristics of the Study Population.

	Blood Type O *n* = 61	Blood Type A, B, AB *n* = 74	*p*-Value
Age (years)	64.61 ± 9.86	62.46 ± 11.8	0.251
Gender (male)	31 (50.8%)	45 (60.8%)	0.175
Operating Room Time (min)	197.7 ± 74	200 ± 74	0.82
Estimated Blood Loss (mL)	474.3 ± 696	527.8 ± 599	0.295
Intraoperative Blood Transfusion ^∞^	15 (24.59%)	21 (28.37%)	0.698
Postoperative Blood Transfusion ^∞^	9 (14.8%)	11 (14.9)	0.906
Size of Largest Lesion (mm)	30.4 ± 24.0	25.3 ± 15.4	0.286
Major Liver Resection ^β^	17 (22.9%)	24 (32.4%)	0.566
Overall Complications	18 (29.5%)	32 (43.2%)	0.088
Grade of Complication ^µ^ 0–2 3 >4	56 (91.8%) 1 (1.6%) 4 (6.6%)	66 (89.1%) 2 (2.7%) 6 (8.2%)	0.899
30 Days Readmission	12 (19.7%)	16 (21.6%)	0.807
Recurrence of Colorectal Cancer	30 (49.1%)	37 (50%)	0.685

^∞^ Patients who received at least one packed red blood cell unit. ^β^ Defined as three or more segmental resections. ^µ^ The severity of complications was graded using the Clavien–Dindo scoring system [28].

**Table 2:** Laboratory Characteristics of the Study Population.

	Blood Type O *n* = 61	Blood Type A, B, AB *n* = 74	*p*-Value
Rh Positive	58 (95.1%)	65 (87.8%)	0.141
Mean Preoperative Hemoglobin (g/dL)	12.7 ± 1.5	12.0 ± 1.4	0.538
Mean Postoperative Day 1 Hemoglobin (g/dL)	11.1 ± 1.4	11.2 ± 1.6	0.517
Mean Lowest Postoperative Hemoglobin (g/dL)	9.5 ± 1.5	9.9 ± 1.4	0.128
Mean Preoperative Platelet (g/uL)	222.5 ± 59.6	211.9 ± 75.1	0.193
Mean Preoperative Operative INR	1.08 ± 0.41	1.06 ± 0.17	0.406
Platelet—Matched to Lowest Hemoglobin (k/uL)	179.9 ± 80.5	171.6 ± 74.7	0.404
Highest Postoperative INR	1.41 ± 0.44	1.66 ± 0.68	0.035
Preoperative White Blood Cells (k/dL)	7.3 ± 2.6	6.7 ± 1.8	0.258
Preoperative CEA (ng/mL)	21.4 ± 34.8	40.9 ± 109.4	0.743
Preoperative CA 19-9 (U/mL)	46.8 ± 79.8	67.0 ± 169.5	0.409
Preoperative Vitamin D (nmol/L)	42.7 ± 26.8	37.4 ± 33.7	0.316
Preoperative Albumin (g/dL)	4.1 ± 0.43	4.1 ± 0.40	0.960

INR: International Normalized Ratio, CEA: carcinoembryonic antigen, CA 19-9: carbohydrate antigen 19-9.

**Table 3:** Preoperative and Postoperative Calculations of Hemoglobin Drop.

	Blood Type O *n* = 61	Blood Type A, B, AB *n* = 74	*p*-Value
All Study Population			
∆Hemoglobin (g/dL) Preoperative and Postoperative Day 1	1.67 ± 1.46	1.74 ± 1.47	0.613
∆Hemoglobin (g/dL) Postoperative Day 1 and Lowest	1.56 ± 0.97	1.30 ± 1.2	0.209
∆Hemoglobin Preoperative and Lowest	3.2 ± 1.7	3.0 ± 1.4	0.582
Patients without Intraoperative Blood Transfusion			
∆Hemoglobin (g/dL) Preoperative and Postoperative Day 1	1.60 ± 1.39	1.68 ± 1.26	0.522
∆Hemoglobin (g/dL) Postoperative Day 1 and Lowest	1.43 ± 0.91	1.28 ± 1.1	0.354
∆Hemoglobin (g/dL) Preoperative and Lowest	3.0 ± 1.7	3.0 ± 1.3	0.853
Patients without Postoperative Blood Transfusion			
∆Hemoglobin (g/dL) Preoperative and Postoperative Day 1	1.53 ± 1.41	1.64 ± 1.21	0.459
∆Hemoglobin (g/dL) Postoperative Day 1 and Lowest	1.51 ± 0.96	1.28 ± 1.2	0.163
∆Hemoglobin (g/dL) Preoperative Operative and Lowest	3.1 ± 1.75	2.9 ± 1.3	0.793
Patients without Intraoperative and Postoperative Blood Transfusion			
∆Hemoglobin (g/dL) Preoperative Operative and Postoperative Day 1	1.48 ± 1.37	1.72 ± 1.23	0.275
∆Hemoglobin (g/dL) Postoperative Day-1 and Lowest	1.46 ± 0.96	1.16 ± 1.02	0.113
∆Hemoglobin (g/dL) Preoperative and Lowest	2.93 ± 1.71	2.9 ± 1.3	0.963

∆: Difference in Hemoglobin Levels.

## Data Availability

The data used in this study were obtained from the hospital’s computerized database and are not publicly available online.

## Abbreviations

vWF	von Willebrand factor
CRLM	colorectal liver metastases
FLR	future liver remnant
IRB	institutional review board
INR	international normalized ratio
CA 19-9	cancer antigen 19-9
CEA	carcinoembryonic antigen
TLV	total liver volume
NCCN	National Comprehensive Cancer Network
CT	computed tomography
Hb	hemoglobin
POD-1	postoperative day 1
EBL	estimated blood loss

## References

[1] Farges O., Goutte N., Bendersky N., Falissard B. (2012). ACHBT-French Hepatectomy Study Group Incidence and risks of liver resection: An all-inclusive French nationwide study. Ann. Surg..

[2] Fong Y., Gonen M., Rubin D., Radzyner M., Brennan M.F. (2005). Long-term survival is superior after resection for cancer in high-volume centers. Ann. Surg..

[3] Nathan H., Cameron J.L., Choti M.A., Schulick R.D., Pawlik T.M. (2009). The volume-outcomes effect in hepato-pancreato-biliary surgery: Hospital versus surgeon contributions and specificity of the relationship. J. Am. Coll. Surg..

[4] Scheele J., Stang R., Altendorf-Hofmann A., Paul M. (1995). Resection of colorectal liver metastases. World J. Surg..

[5] Hasegawa K., Kokudo N., Imamura H., Matsuyama Y., Aoki T., Minagawa M., Sano K., Sugawara Y., Takayama T., Makuuchi M. (2005). Prognostic impact of anatomic resection for hepatocellular carcinoma. Ann. Surg..

[6] Colli J.L., Colli A. (2006). International comparisons of prostate cancer mortality rates with dietary practices and sunlight levels. Urol. Oncol..

[7] Kobayashi M., Ikeda K., Hosaka T., Sezaki H., Someya T., Akuta N., Suzuki F., Suzuki Y., Saitoh S., Arase Y. (2006). Dysplastic nodules frequently develop into hepatocellular carcinoma in patients with chronic viral hepatitis and cirrhosis. Cancer.

[8] Shimada M., Matsumata T., Akazawa K., Kamakura T., Itasaka H., Sugimachi K., Nose Y. (1994). Estimation of risk of major complications after hepatic resection. Am. J. Surg..

[9] Meijer C., Wiezer M.J., Hack C.E., Boelens P.G., Wedel N.I., Meijer S., Nijveldt R.J., Statius Muller M.G., Wiggers T., Zoetmulder F.A. (2001). Coagulopathy following major liver resection: The effect of rBPI21 and the role of decreased synthesis of regulating proteins by the liver. Shock.

[10] Tanabe G., Sakamoto M., Akazawa K., Kurita K., Hamanoue M., Ueno S., Kobayashi Y., Mitue S., Ogura Y., Yoshidome N. (1995). Intraoperative risk factors associated with hepatic resection. Br. J. Surg..

[11] Franchini M., Rossi C., Mengoli C., Meschieri M., Frattini F., Crestani S., Giacomini I., Luppi M., Bonfanti C. (2013). O blood group is a risk factor for severe mucosal hemorrhage in orally anticoagulated patients. J. Thromb. Thrombolysis.

[12] Franchini M., Capra F., Targher G., Montagnana M., Lippi G. (2007). Relationship between ABO blood group and von Willebrand factor levels: From biology to clinical implications. Thromb. J..

[13] Assaf W., Wattad A., Ali-Saleh M., Shalabna E., Lavie O., Abramov Y. (2024). Evaluation of blood type as a potential risk factor for hemorrhage during vaginal hysterectomy. Eur. J. Obstet. Gynecol. Reprod. Biol..

[14] Leonard D.S., Fenton J.E., Hone S. (2010). ABO blood type as a risk factor for secondary post-tonsillectomy haemorrhage. Int. J. Pediatr. Otorhinolaryngol..

[15] Cortes G.A., Moore M.J., El-Nakeep S. (2025). Physiology, von willebrand factor. StatPearls.

[16] Dentali F., Sironi A.P., Ageno W., Bonfanti C., Crestani S., Frattini F., Steidl L., Franchini M. (2013). Relationship between ABO blood group and hemorrhage: A systematic literature review and meta-analysis. Semin. Thromb. Hemost..

[17] Horwich L., Evans D.A., McConnell R.B., Donohoe W.T. (1966). ABO blood groups in gastric bleeding. Gut.

[18] Evans D.A., Horwich L., McConnell R.B., Bullen M.F. (1968). Influence of the ABO blood groups and secretor status on bleeding and on perforation of duodenal ulcer. Gut.

[19] Ionescu D.A., Marcu I., Bicescu E. (1976). Cerebral thrombosis, cerebral haemorrhage, and ABO blood-groups. Lancet.

[20] DeBot M., Eitel A.P., Moore E.E., Sauaia A., Lutz P., Schaid T.R., Hadley J.B., Kissau D.J., Cohen M.J., Kelher M.R. (2022). Blood type o is a risk factor for hyperfibrinolysis and massive transfusion after severe injury. Shock.

[21] Ward S.E., O’Sullivan J.M., O’Donnell J.S. (2020). The relationship between ABO blood group, von Willebrand factor, and primary hemostasis. Blood.

[22] Maezawa K., Nozawa M., Gomi M., Sugimoto M., Maruyama Y. (2021). Association of ABO blood group with postoperative total bleeding volume in patients undergoing total hip arthroplasty. Vox Sang..

[23] Archer N.M., Forbes P.W., Dargie J., Manganella J., Licameli G.R., Kenna M.A., Brugnara C. (2020). Association of blood type with postsurgical mucosal bleeding in pediatric patients undergoing tonsillectomy with or without adenoidectomy. JAMA Netw. Open.

[24] Welsby I.J., Jones R., Pylman J., Mark J.B., Brudney C.S., Phillips-Bute B., Mathew J.P., Campbell M.L., Stafford-Smith M. (2007). Cardiothoracic Anesthesiology Research Endeavours (C.A.R.E.), Department of Anesthesiology, Duke University Medical Center ABO blood group and bleeding after coronary artery bypass graft surgery. Blood Coagul. Fibrinolysis.

[25] Entezari P., Toskich B.B., Kim E., Padia S., Christopher D., Sher A., Thornburg B., Hohlastos E.S., Salem R., Collins J.D. (2022). Promoting Surgical Resection through Future Liver Remnant Hypertrophy. Radiographics.

[26] Adams R.B., Aloia T.A., Loyer E., Pawlik T.M., Taouli B., Vauthey J.-N., Americas Hepato-Pancreato-Biliary Association, Society of Surgical Oncology, Society for Surgery of the Alimentary Tract (2013). Selection for hepatic resection of colorectal liver metastases: Expert consensus statement. HPB.

[27] Shindoh J., Tzeng C.-W.D., Aloia T.A., Curley S.A., Zimmitti G., Wei S.H., Huang S.Y., Mahvash A., Gupta S., Wallace M.J. (2013). Optimal future liver remnant in patients treated with extensive preoperative chemotherapy for colorectal liver metastases. Ann. Surg. Oncol..

[28] Dindo D., Demartines N., Clavien P.-A. (2004). Classification of surgical complications: A new proposal with evaluation in a cohort of 6336 patients and results of a survey. Ann. Surg..

[29] Bird G.W., Wingham J., Watkins W.M., Greenwell P., Cameron A.H. (1978). Inherited “mosaicism” within the ABO blood group system. J. Immunogenet..

[30] Magen David Adom in Israel (2022). Blood Type Distribution in Israel. https://www.mdais.org.

[31] Sun W., Wen C.-P., Lin J., Wen C., Pu X., Huang M., Tsai M.K., Tsao C.K., Wu X., Chow W.-H. (2015). ABO blood types and cancer risk--a cohort study of 339,432 subjects in Taiwan. Cancer Epidemiol..

[32] Cui H., Qu Y., Zhang L., Zhang W., Yan P., Yang C., Zhang M., Bai Y., Tang M., Wang Y. (2023). Epidemiological and genetic evidence for the relationship between ABO blood group and human cancer. Int. J. Cancer.

[33] Moeller A., Weippert-Kretschmer M., Prinz H., Kretschmer V. (2001). Influence of ABO blood groups on primary hemostasis. Transfusion.

[34] Gill J.C., Endres-Brooks J., Bauer P.J., Marks W.J., Montgomery R.R. (1987). The effect of ABO blood group on the diagnosis of von Willebrand disease. Blood.

[35] Sadler J.E., Mannucci P.M., Berntorp E., Bochkov N., Boulyjenkov V., Ginsburg D., Meyer D., Peake I., Rodeghiero F., Srivastava A. (2000). Impact, diagnosis and treatment of von Willebrand disease. Thromb. Haemost..

[36] Wright J.D., Herzog T.J., Tsui J., Ananth C.V., Lewin S.N., Lu Y.-S., Neugut A.I., Hershman D.L. (2013). Nationwide trends in the performance of inpatient hysterectomy in the United States. Obstet. Gynecol..

[37] Miller C.H., Haff E., Platt S.J., Rawlins P., Drews C.D., Dilley A.B., Evatt B. (2003). Measurement of von Willebrand factor activity: Relative effects of ABO blood type and race. J. Thromb. Haemost..

[38] Blustin J.M., McBane R.D., Mazur M., Ammash N., Sochor O., Grill D.E., Wysokinski W.E. (2015). The association between thromboembolic complications and blood group in patients with atrial fibrillation. Mayo Clin. Proc..

[39] Orstavik K.H., Magnus P., Reisner H., Berg K., Graham J.B., Nance W. (1985). Factor VIII and factor IX in a twin population. Evidence for a major effect of ABO locus on factor VIII level. Am. J. Hum. Genet..

[40] Larsen T.B., Johnsen S.P., Gislum M., Møller C.A.I., Larsen H., Sørensen H.T. (2005). ABO blood groups and risk of venous thromboembolism during pregnancy and the puerperium. A population-based, nested case-control study. J. Thromb. Haemost..

[41] Jick H., Slone D., Westerholm B., Inman W.H., Vessey M.P., Shapiro S., Lewis G.P., Worcester J. (1969). Venous thromboembolic disease and ABO blood type. A cooperative study. Lancet.

[42] Talbot S., Wakley E.J., Ryrie D., Langman M.J. (1970). ABO blood-groups and venous thromboembolic disease. Lancet.

[43] Reddy V.M., Daniel M., Bright E., Broad S.R., Moir A.A. (2008). Is there an association between blood group O and epistaxis?. J. Laryngol. Otol..

[44] Drukker L., Srebnik N., Elstein D., Levitt L., Samueloff A., Farkash R., Grisaru-Granovsky S., Sela H.Y. (2016). The association between ABO blood group and obstetric hemorrhage. J. Thromb. Thrombolysis.

[45] Kahr M.K., Franke D., Brun R., Wisser J., Zimmermann R., Haslinger C. (2018). Blood group O: A novel risk factor for increased postpartum blood loss?. Haemophilia.

[46] Tatar Aksoy H., Eras Z., Canpolat F.E., Dilmen U. (2013). The association between neonatal ABO blood group and intraventricular hemorrhage in extremely low birth weight infants. J. Thromb. Haemost..

[47] Halonen P., Linko K., Wirtavuori K., Hästbacka J., Ikkala E. (1987). Evaluation of risk factors in intraoperative bleeding tendency. Ann. Chir. Gynaecol..

[48] Ali-Saleh M., Lavie O., Abramov Y. (2019). Evaluation of blood type as a potential risk factor for early postpartum hemorrhage. PLoS ONE.

[49] Shirabe K., Kajiyama K., Harimoto N., Tsujita E., Wakiyama S., Maehara Y. (2010). Risk factors for massive bleeding during major hepatectomy. World J. Surg..

[50] Helling T.S., Blondeau B., Wittek B.J. (2004). Perioperative factors and outcome associated with massive blood loss during major liver resections. HPB.

[51] McNally S.J., Revie E.J., Massie L.J., McKeown D.W., Parks R.W., Garden O.J., Wigmore S.J. (2012). Factors in perioperative care that determine blood loss in liver surgery. HPB.

[52] Abu Hilal M., Underwood T., Taylor M.G., Hamdan K., Elberm H., Pearce N.W. (2010). Bleeding and hemostasis in laparoscopic liver surgery. Surg. Endosc..

